# Contralateral Cochlear Labyrinthine Concussion without Temporal Bone Fracture: Unusual Posttraumatic Consequence

**DOI:** 10.1155/2016/2123182

**Published:** 2016-09-21

**Authors:** I. M. Villarreal, D. Méndez, J. M. Duque Silva, P. Ortega del Álamo

**Affiliations:** Otorhinolaryngology Department, “Móstoles” University Hospital, Madrid, Spain

## Abstract

*Introduction*. Labyrinthine concussion is a term used to describe a rare cause of sensorineural hearing loss with or without vestibular symptoms occurring after head trauma. Isolated damage to the inner ear without involving the vestibular organ would be designated as a cochlear labyrinthine concussion. Hearing loss is not a rare finding in head trauma that involves petrous bone fractures. Nevertheless it generally occurs ipsilateral to the side of the head injury and extraordinarily in the contralateral side and moreover without the presence of a fracture.* Case Report*. The present case describes a 37-year-old patient with sensorineural hearing loss and tinnitus in his right ear after a blunt head trauma of the left-sided temporal bone (contralateral). Otoscopy and radiological images showed no fractures or any abnormalities. A severe sensorineural hearing loss was found in his right ear with a normal hearing of the left side.* Conclusion*. The temporal bone trauma requires a complete diagnostic battery which includes a neurotologic examination and a high resolution computed tomography scan in the first place. Hearing loss after a head injury extraordinarily occurs in the contralateral side of the trauma as what happened in our case. In addition, the absence of fractures makes this phenomenon even more unusual.

## 1. Introduction

Labyrinthine concussion is a term used to depict a sensorineural hearing loss (SNHL) with or without vestibular symptoms occurring after head trauma. The temporal bone is at risk for injury in the setting of high-impact craniofacial trauma. Skull fractures due to blunt force trauma occur in 3% to 22% of patients with head trauma. 80% to 90% of cases with temporal bone trauma sustain unilateral injury [1–3]. Hearing loss secondary to head trauma, especially if a temporal bone fracture is associated, is not an unusual clinical consequence. There are several theories postulated to explain the phenomenon of deafness after head injury. Nevertheless, hearing loss in the contralateral side of the injury without evidence of a skull base fracture is a very exceptional finding [4, 5].

## 2. Clinical Case

We report a case of a 37-year-old male patient who presented to the emergency room with a left side head trauma after being hit with a hammer during an altercation. He complained of contralateral hearing loss and tinnitus denying any right side injury. No vertigo accompanied his deafness. The onset of the symptoms was immediately after the head trauma.

The patient had no personal or family history of previous hearing disorders, including hearing loss or anatomical alterations; hence we believe he had a normal hearing prior to trauma since we have no previous audiological records.

**Figure 1:**
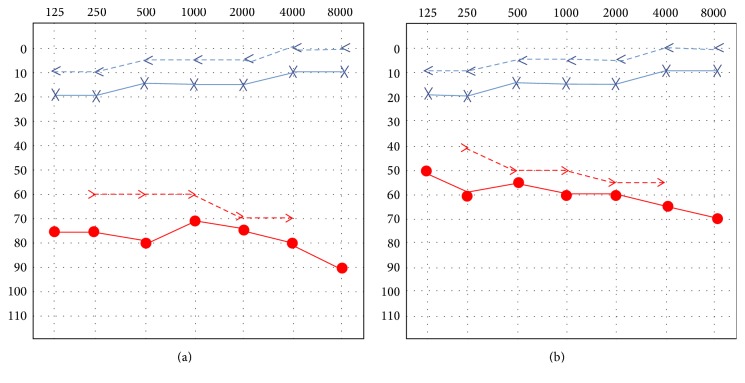
Pure tone audiometry: (a) Severe sensorineural hearing loss in the right ear and normal hearing in his left ear. (b) Treatment with oral corticosteroids was administered gaining approximately 15 dB in all frequencies after one week.

**Figure 2:**
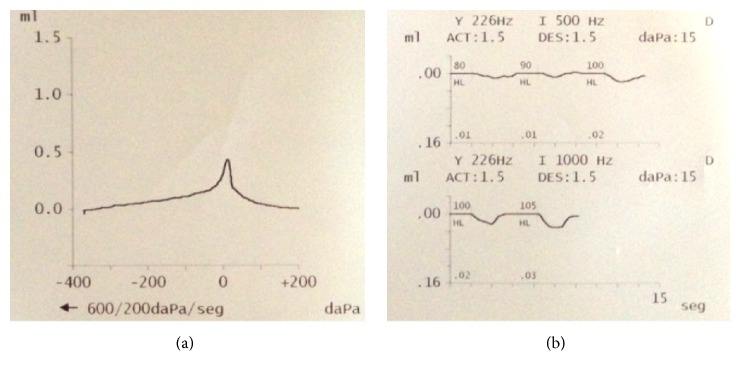
(a) Right ear tympanogram was consistent with a type A curve; (b) acoustic reflex was present.

**Figure 3:**
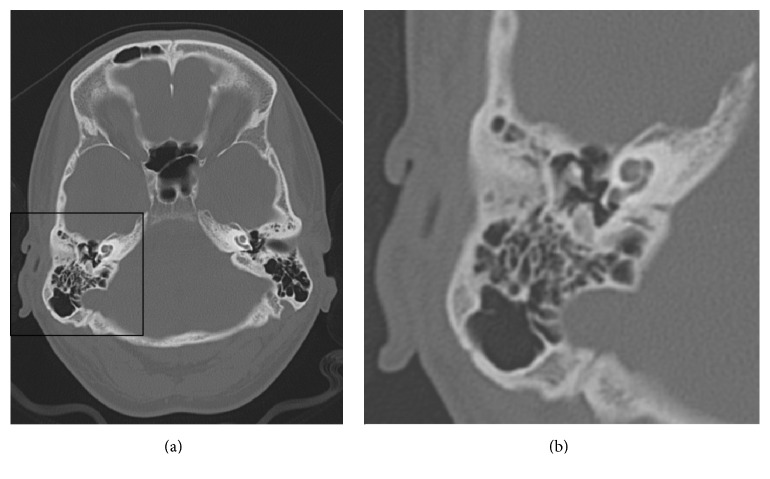
Computed tomography (CT) scan (axial section): No radiological evidence of temporal bone fracture, intact ossicular chains, no hemorrhages, and no obvious fistulas.

Otoscopic examination revealed normal bilateral intact tympanic membranes. A positive Rinne test in both ears and a Weber test lateralized to the left side in 256, 512, and 1024 Hz. Severe sensorineural hearing loss in the right ear and normal hearing in his left ear were demonstrated with a pure tone audiometry test (Figure 1(a)). Additionally, a tympanogram of his right ear was consistent with a type A pattern and a proper acoustic reflex was present (Figure 2). Regarding imaging studies, a high definition computed tomography (CT) scan showed no radiological evidence of temporal bone fractures, intact ossicular chains, no hemorrhages, and no obvious fistulas (Figure 3). No vestibular otoneurological tests were performed due to the lack of vestibular symptoms.

Treatment with oral corticosteroids (Deflazacort at a 1 mg/kg per day dose beginning with 70 mg and slowly tapering the dose) was administered gaining 15 dB in all frequencies within a week when follow-up was assessed. (Figure 1(b)). The patient refused to receive intravenous treatment and only accepted oral medication. The patient made only one follow-up visit in our department and moved out of town afterwards.

## 3. Discussion

Temporal bone fractures due to blunt head trauma are not an unusual finding. They are classified according to their anatomical orientation and moreover correlated with their clinical presentation. The three possibilities involve a longitudinal, a transversal, or a mixed type of fracture. These fractures may involve some complications or sequelae such as facial paralysis, conductive hearing loss, cerebrospinal fluid leakage (CSF), SNHL, vertigo, and vascular injury [2, 3]. Temporal fractures should be well recognized. A setback in the diagnosis of a temporal bone fracture may lead to a delay in the correct surgical treatment when needed (such as cases of a complete facial paralysis) or a conservative treatment with a strict vigilance in cases of CSF leak.

Longitudinal fractures account for the majority of the cases involving usually a conductive hearing loss and in very few cases facial paralysis or vertigo. Some studies have reported the mixed type of hearing loss as the most common. These fractures are usually associated with temporal or parietal bone trauma. Transversal fractures have a major incidence of facial paralysis and if accompanied by hearing loss it usually involves a sensorineural type due to its labyrinth involvement [1–3]. Nevertheless, since the arrival of high definition CT scan alternative classifications for temporal bone fractures have been proposed. Kelly and Tami suggested that the involvement of the otic capsule should be taken into account [4]. Otic capsule violating injuries have a higher incidence of CSF leak [2]. Some authors have classified them as petrous fractures or those involving the otic capsule or petrous apex and nonpetrous fractures which are subcategorized into middle ear involvement or mastoid involvement [1–3]. This classification according to the authors allows a better correlation regarding the related clinical findings.

Hearing loss is not a rare finding in head trauma that involves petrous bone fractures. It generally occurs ipsilateral to the side of the head injury. Hearing loss extraordinarily occurs in the contralateral side and moreover without the presence of a fracture as what happens in our case [5].

Several theories have been proposed to explain this circumstance such as labyrinthine concussion resulting from a fracture to the bony labyrinthine capsule. However the mechanism involving this phenomenon remains unclear. Labyrinthine concussion is a term used to depict a SNHL with or without vestibular symptoms occurring after head trauma [5–7]. A subclassification can be made regarding labyrinthine concussion. If there is an isolated damage to the inner ear it would be a cochlear labyrinthine concussion and if the damage involves the otolith organ a vestibular labyrinthine concussion would be the preferable designation [8, 9]. Labyrinthine concussion in most cases involves a SNHL with a notch in the 4–6 kHz resembling acoustic trauma, positional vertigo, or tinnitus [7, 8, 10]. Most cases show an accompanying tinnitus regardless of the presence of vertigo as what happened to our patient.

Disruption to the organ of Corti has been suggested involving a pattern seen when high-pressure waves of the intracranial CSF caused by intense airborne sounds transmitted to the cochlea. In the animal experiments from which this theory was built, hemorrhage sites and microcirculation disturbances in the cochlea destroying the sensory epithelium due to rupture of vessels in the membranous labyrinth were also seen [6, 7, 10]. Histopathological changes reported in cochlear labyrinthine concussion (cochlear concussion) vary from mild alterations in the internal or external hair cells to a complete degeneration of the organ of Corti. In cochlear labyrinthine concussion basilar membrane shearing and eventual auditory nerve fiber avulsion might also be described. Another hypothesis involves direct disruption of the membranous labyrinth with inflammatory changes resulting in fibrotic tissue and scarring accumulation and new bone formation. These changes are possible causes of a vestibular labyrinthine concussion [6, 7, 10].

One differential diagnosis may include a perilymph fistula but in this case we would expect the hearing loss to be accompanied not only by tinnitus but also by vestibular symptoms. This did not occur in our case [1, 6, 7]. A benign paroxysmal positional vertigo may also result from head trauma but vertigo must be present as the name indicates and no hearing loss is seen [7]. Another possibility is an isolated eighth nerve stretch injury which may be assessed by tests such as vestibular evoked myogenic potentials (VEMPs) or a severe crush injury or nerve transaction which is extremely rare and might be evaluated with a “promontory examination” test or a brainstem evoked response audiometry (BERA) [9, 10].

We have found only one case described similar to our case except for vestibular symptoms accompanying the SNHL [6]. Three cases of labyrinthine concussion characterized by SNHL in the contralateral side of the head injury without vestibular symptoms have been described [7]. Nevertheless, they were accompanied by fracture of the ipsilateral side making these cases different from ours. On the other hand, Chiaramonte et al. [9] presented a case of a patient with bilateral SNHL without temporal bone fractures but with the presence of severe vertigo making it different from our case based on these two details. Two other cases of head trauma without cranial base fractures with SNHL were reported but no details regarding other symptoms such as vertigo are described in these 2 specific cases nor if the SNHL was contralateral [11].

## 4. Conclusion

Temporal bone trauma requires a complete diagnostic battery which includes a neurotologic examination, a high resolution CT scan (in cases with clinical suspicion of temporal fracture), an electroneuronography if facial paralysis is present, audiometric tests, and vestibular tests if required [7].

There is no definitive treatment for labyrinthine concussion. Corticosteroids are controversial and most of them are managed expectantly. We administered steroids as described before to our patient and there was 15 dB improvement in all frequencies. Nevertheless, we have no evidence that the hearing improvement was a response to corticotherapy or it just occurred incidentally. Further studies have to be performed to prove the effectiveness of this treatment [12]. The prognosis of hearing recovery is disappointing in most cases.

Hearing loss after a head injury extraordinarily occurs in the contralateral side of the trauma as what happened in our case and moreover in the absence of fractures which makes this phenomenon even more unusual.
